# Factors associated with acculturative stress among international medical students in an Egyptian university

**DOI:** 10.1186/s12909-024-05947-5

**Published:** 2024-09-15

**Authors:** Yomna Ayman Ali, Rasha Saad Hussein, Nayera Samy Mostafa, Ayat Farouk Manzour

**Affiliations:** Department of Community, Environmental and Occupational Medicine, Faculty of Medicine, Ain Shams University, Cairo, Egypt

**Keywords:** Acculturation, Acculturative stress, International students, Medical study

## Abstract

**Background:**

International students frequently face difficulties that are specific to the typical college experience, in addition to facing other factors of acculturative stress, including cultural differences, language barriers, and comfortability in accommodation.

**Aim:**

The study aims to measure the prevalence of acculturative stress among first-year international students enrolled at Faculty of Medicine in an Egyptian University and aims to reveal the factors that are associated with acculturative stress in these students.

**Methodology:**

A cross-sectional study was conducted on 422 international students in the academic year 2021–2022, using the Acculturative Stress Scale for International Students (ASSIS). The factors associated with acculturative stress were examined using the following criteria: comfort in accommodation, student adjustment to college, language proficiency, and academic pressure.

**Results:**

This study showed that 28.7% of the international students were “above the warning sign” of acculturative stress (above 109). The major stressors of acculturative stress among international students were the non-specific concerns, homesickness, and perceived discrimination, while the least reported stressors were fear and guilt. International students who scored in the “above the warning sign” in the (ASSIS) were mostly females (35.3%) and non-Arab students (37.8%). Having a friend or a family member living in Egypt significantly reduced the level of acculturative stress. Moreover, the ASSIS total score showed a negative correlation with the scores for overall language proficiency, comfort in accommodation, academic adjustment, and student adjustment to college.

**Conclusion:**

Acculturative stress among the studied groups is influenced by several factors, including nationality, English and Arabic language proficiency, academic adjustment, and comfort of living. That being said, the most significant stressor is the country of origin, which is defined in terms of nationality or language proficiency. Consequently, resources that ensure the sustainability and growth of international students throughout their educational process must be made available to a multicultural environment in order to support and retain those students.

**Clinical trial number:**

Not applicable.

**Supplementary Information:**

The online version contains supplementary material available at 10.1186/s12909-024-05947-5.

## Introduction

Egypt has lately drawn a lot of international students, especially those who want to study medicine. That being said, the decision to study abroad has a significant impact on the student’s stress, and there is a lot to be considerd before choosing to study abroad. This stress may be related to the difficulty and demands of the medicine field itself or may be related to how the student finds it challenging to integrate into the local community because of the acculturation process [1–4].

Every medical student experiences a variety of stressors, including psychological stress from exams, social pressure, long faculty duration, challenging course material, and personal issues. In addition to studying medicine, overseas students encounter an entirely distinct set of challenges, including linguistic and cultural hurdles, different study systems, academic difficulties, and financial difficulties. The students’ concerns about living comfortably in the dorms and their happiness with other college students and co-workers are also on their minds. As a result, individuals are more vulnerable to feeling acculturative stress, which can have negative effects on their mental and psychological well-being, leading to somatization disorders, despair, and anxiety [5, 6].

As becoming an immigrant can be viewed as a stressful experience, the phrase “acculturative stress” is frequently utilized to describe the unique challenges associated with immigration [2, 7].

Most of the tools developed for acculturation research evaluate acculturation in a broad sense. However, other measures that are specifically designed to assess the acculturative stress experienced by students studying abroad exist. First, Sandhu and Asrabadi (1994) created the Acculturative Stress Scale for International Students (ASSIS).

A higher score on the scale denotes a greater degree of acculturative stress. The measure’s total score ranges from 36 to 180. The authors established a threshold level for counseling and psychological assistance at 109, which is two standard deviations above the mean score of 66.32 [8]. The Index of Life Stress for Asian Students (ILS), created by Yang and Clum (1995), is the second scale that was used. General stress and acculturative stress are not distinguished by this scale. Rather, the scale evaluates the overall stress level among Asian students. The ILS has 31 items covering five stressor categories (language barrier, cultural adjustment, academic worries, financial issues, and future outlook) that were determined by a review of the literature.

Higher scores indicate higher levels of stress in life. The questions are on a 4-point Likert scale, with 0 representing never, and 3 representing often. The stress levels in the authors’ sample and the normative data of American college students differed. Therefore, it is possible that this measure is not a good way to identify acculturative stress [9].

The Acculturative Hassles Scale for Chinese Students (AHSCS), created by Pan and her colleagues (2008), is the third and most recent scale that was used. The Acculturative Stressor Scale for Chinese Students (ASSCS), an 18-item measure with four components (language insufficiency, cultural difference, academic work, and social interaction) was initially described as the precursor to the AHSCS. As it is in Chinese, the AHSCS is more pertinent to Chinese students [10].

All things considered, the Acculturative Stress Scale for International Students (ASSIS) is the best instrument to be employed in the current study as it is more objective, thorough, and unaffected by cultural variations.

The ASSIS scale was utilized in a prior study involving Pakistani students in Chinese universities. The results indicated that a large percentage of the studied group (68.53%) experienced acculturative stress. Perceived discrimination was the most affected domain [11] in the study.

Previous research on the risk factors of acculturative stress has demonstrated that a wide range of factors are connected to the experience. Language obstacles, cultural diversity, personality, and inclusion in society are a few of these aforementioned factors. The best-documented factors include age, gender, and language proficiency [4, 12].

Higher levels of English proficiency have also been linked to reduced levels of academic stress. According to a research on the relationship between acculturative stress and English language competency among Chinese students studying in the U.S., it may be more difficult for Chinese students to interact socially. They also might find difficulty in seeking help if they are unable to communicate in English. Thus, this could be detrimental to their psychological health [13, 14].

Additionally, sociocultural pressures (including money problems, housing issues, and transportation) provide another obstacle for international students [2].

Notably, the more information the faculty administration obtains about the stressors and factors related to the acculturation process, the more it can create more successful programs to assist newcomers in the faculty through institutional, academic, social, psychological, and linguistic interventions [4, 15].

Within months of enrolling at the university, the majority of international first-year students at the Faculty of Medicine pass the honeymoon phase and move into the shock period. Based on this, the purpose of this research is to determine the prevalence of acculturative stress in first-year international students and to pinpoint its contributing elements in order to develop programs that will be effective in assisting students with their sociocultural transition [2].

### Research knowledge gaps

Adaptation to university life is a challenging process, especially in studying medicine. To facilitate and improve the adaptation process of undergraduate medical students during their university life, different stressors and challenges should be addressed to create an effective academic and social international environment.

### Research objectives

The research objectives are: to measure the prevalence of acculturative stress among international students, and to identify the factors that are associated with it.

## Materials and methods

A cross-sectional study was conducted from November 2021 to November 2022. The study includes international students who were in their first year (the academic year 2021–2022) at the Faculty of Medicine in one of the oldest Egyptian universities.

### Sample size

The sample size was calculated by using the Epi Info 7 program for sample size calculation. Reviewing results from previous relevant studies (Zhang and Brunton 2007) showed that about 55% of international university students got exposed to socio-cultural stressors with a margin of error = 5%, a 95% confidence level, and a 10% to compensate the incomplete data. A sample size of at least 418 students was therefore needed for the quantitative study.

### Sampling method

**Fig. 1:**
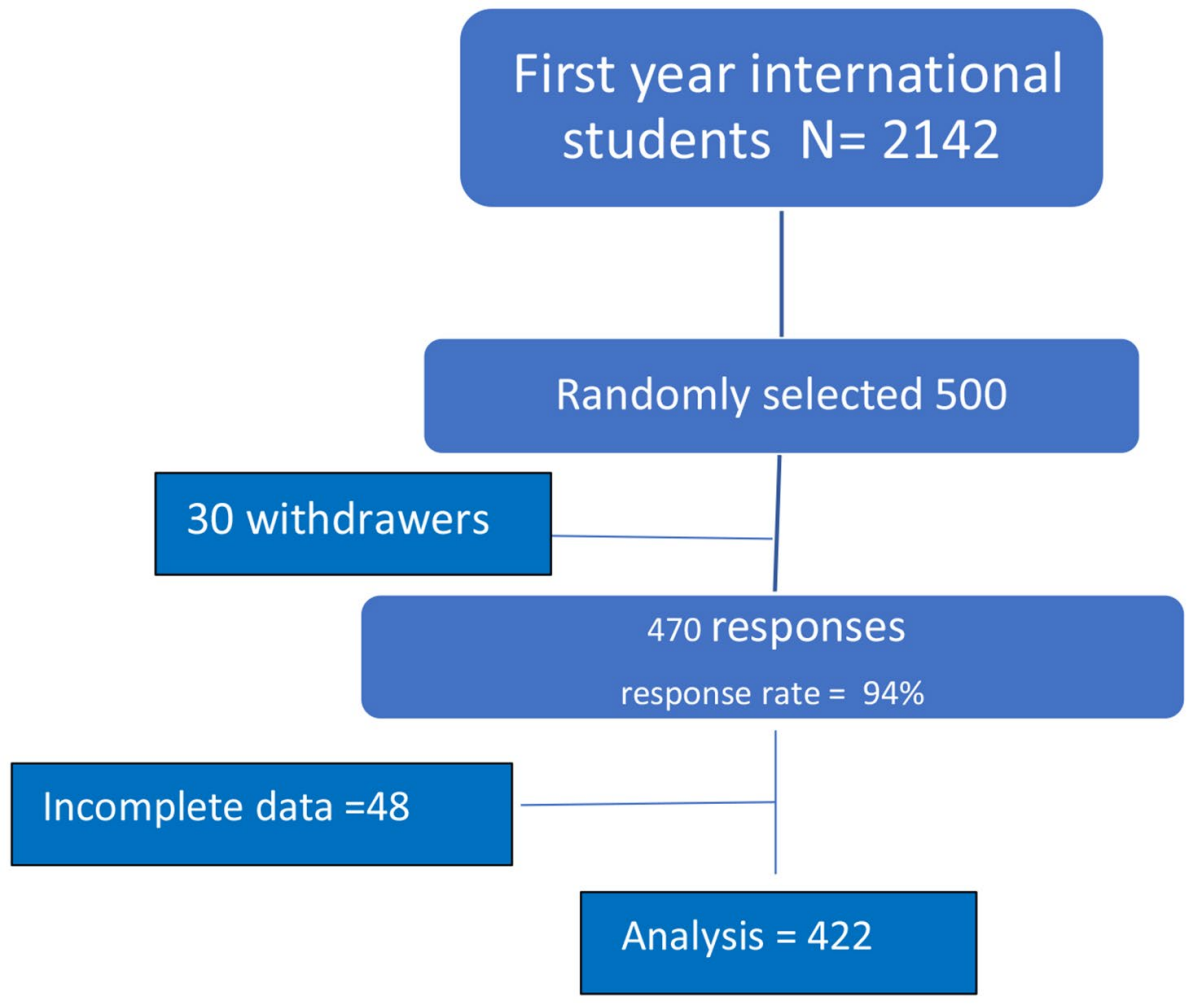
Random Selection of First-Year International Students

A simple random sample method was used, using a computer random number generator after entering the students I.D. numbers that were obtained from student affairs department (Fig. 1).

### The pilot phase

The pilot study was carried out to test the understanding of the tool by some of the study participants. The self-administered questionnaire was tested on 42 students (10% of the study sample size) before conducting the study. No questions were changed after the pilot. This showed that the tool was suitable and understood by the students. Hence, no changes were required to be done to the questionnaire. The data collected during the pilot was added to the total required sample size.

## Study tools

### A self-administered questionnaire in English was used and included the following sections

#### Sociodemographic characteristic data [2, 2–4]

i.e. age; gender; and nationality; whether one of the relatives is a physician or not; whether the student speaks Arabic or not; the presence of a friend or a family member who is living in Egypt; the place of accomodation; and source of financial support.

#### Comfortability at the accommodation [2–4]

Using a self-rated scale, the degree of comfort with the food, transportation, climate, and dorm life was assessed. Participants indicated how they felt on a Likert-type scale ranging from 1 (not comfortable) to 5 (very comfortable). The sum of the values for each response was used to determine the score.

#### Acculturative stress scale for international students (ASSIS) [2–8]

The 36 components that make up ASSIS are related to the stress that comes with adjusting to a new culture. A Likert scale ranging from 1 (strong disagreement) to 5 (strong agreement) constituted the replies. The items were sub-divided into 7 subscales:

- Perceived discrimination (8 items).
- Homesickness (4 items).
- Perceived hate/rejection (5 items).
- fear (4 items).
- Stress due to change/culture shock (3 items).
- Guilt (2 items).
- Non-specified concerns (10 items).
- The ASSIS score was calculated by summating the values of each response in all subscales, and the total score was then calculated by calculating the total of all subscales. The total scores range from 36 to 180; higher scores indicate higher levels of stress. The total score of acculturative stress was also categorized according to the warning sign stated in the previous literature [8]. The first category was equal to or below the warning sign, and the second category was above the warning sign. The cut-off level was settled at 104 for identification of acculturative stress [8].

#### Student Adjustment to College [2–16]

The social adjustment subscale, which measures how well students are able to handle the interpersonal and societal pressures of college life, contains questions that were extracted. Using a Likert-type scale, the participants’ responses ranged from 1 (strongly disagree) to 5 (strongly agree). The responses to questions 4 and 6 were reverse coded, and the score was determined by adding the values of each response.

#### Language deficiency and academic pressure: [10]

The final two parts to the questionnaire were taken from the Chinese students’ Acculturative Stressor Scale (ASSCS). On a Likert-type scale ranging from 1 (never) to 5 (always), participants provided their responses. Each response value was added together to determine the score, with the items being reverse coded prior to calculation.

### Data management

The Statistical Package for Social Sciences (SPSS version 25) was used to analyze the data. For all the qualitative data, descriptive analyses were carried out using numbers and percentages. As for the quantitative data, the analyses were carried out using the mean ± SD. All score calculations depended on mathematical summation.

Student t-test and chi-square were conducted to test the bivariate associations for quantitative and qualitative variables, respectively. The Pearson Correlation Analysis was used to explore the correlation between ASSIS and other scores. Linear regression was conducted with acculturative stress total score as the dependent variable and various independent variables extracted from previous literature, entered through the “enter method.” A significance level of *p* < 0.05 was considered statistically significant.

## Results

A total of 422 international students were included in the study. The mean age of the study participants was 19.46 (± 1.64) which is expected as they are from the same study year. Most of the study participants were from Arab countries (71.80%), mostly from Sudan, Palestine, Yemen, and Jordan-which are Arab countries geographically near Egypt. 28.20% of the participants were non-Arabs mostly from India, Nigeria, and South Sudan. Moreover, most of the study participants (74.64%) were living in rented apartments. Regarding the source of their financial support, 371 participants (87.91%) reported that they depended on their parents\family; 53 participants (12.56%) had scholarships; and 2.13% of the participants depended on loans and personal savings (Table 1).

**Table 1:** Participant characteristics (*N* = 422)

Participant Characteristics		Total no. (%)
Age (mean ± SD)	19.46 ± 1.64	
Gender	Male	232 (54.98)
	Female	190 (45.02)
Nationality	Arab Country	303 (71.80)
	Non-Arab Country	119 (28.20)
Having a sibling or other relative a physician	Yes	194 (45.97)
Having a friend, or a family member living in Egypt	Yes	306 (72.51)
Place of Accommodation	University Dorms	65 (15.41)
	Rented Hostel	10 (2.37)
	Rented Apartment	315 (74.64)
	Others*	32 (7.58)
Your score was considered in your country	Low Score	6 (1.46)
	Average Score	131 (31)
	High Score	285(67.54)
Sources of Financial Support	Parents\Family	371 (87.92)
	Scholarship	53 (12.56)
	Loans/Personal Savings.	12 (2.84)
	Others	9 (2.13)

**#**Multiple answers were allowed

**Table 2:** Descriptive statistics of ASSIS total score and Subscales (*N* = 422)

Item (Standard Minimum - Standard Maximum)	Mean ± SD	Median (IQR)	Range
Acculturative Stress Total Score (36–180)	97.4 ± 21.83	98 (82–112)	(38–172)
Perceived Discrimination (8–40)	20.35 ± 6.40	20 (16–24)	(8–40)
Homesickness (4–20)	14.54 ± 3.55	15 (12–17)	(4–20)
Perceived Hate/Rejection (5–25)	12.92 ± 4.45	13 (10–16)	(5–25)
Fear (4–20)	9.13 ± 3.47	9 (7–12)	(4–20)
Stress due to Change/Culture Shock (3–15)	9.25 ± 2.77	9 (7–11)	(3–15)
Guilt (2–10)	5.19 ± 2.17	5 (4–7)	(2–10)
Non-Specific Concerns (10–20)	26.05 ± 7.18	26 (21–31)	(10–50)

The ASSIS mean score was 97.4 ± 21.83. The perceived discrimination subscale ranged from 8 to 40; the mean of this subscale in the present study was 20.35 ± 6.40; the mean of non-specific concerns was 26.05 ± 7.18; and the mean of homesickness was 14.54 ± 3.55 as shown in Table 2.

Numerous significant negative correlations between the studied variables were found by the correlation analysis results. However, the value of the negative correlation ranged from small (*r* = -0.037) to moderate (*r* = -0.351).

Furthermore, the ASSIS total score correlated negatively with the total language deficiency score, the total comfortability of living in the place score, the total academic adjustment score, and the total student adjustment to college score as shown in Table 3.

**Table 3:** Correlation between ASSIS (total score and subscales) and some study variables

Study Variables	ASSIS (Total score)
Total Language Deficiency Score (12–60)	-0.252**
Total comfortability of living in the accommodation place score (10–50)	-0.257**
Total Academic Adjustment Score (11–55)	-0.338**
Total Student Adjustment to College Score (8–40)	-0.240**

*P value ≤ 0.05, ** P value ≤ 0.001

**Table 4:** Bivariate Association of Different Factors and occurrence of ASSIS

Study Characteristics	Acculturative stress			
	≤ warning sign	> warning sign	Test value	*P*-value
	*n* = 301	*n* = 121		
**Age (mean ± SD)**	19.40 ± 1.61	19.62 ± 1.72	t= -1.23	0.218
**Gender**			**X**^**2**^ **=** 7.34	0.007*
Male	178 (76.7)	54 (23.3)		
Female	123 (64.7)	67 (35.3)		
**Nationality**			**x**^**2**^ **=** 6.77	0.009*
Arab Country	227 (74.9)	76 (25.1)		
Non-Arab Country	74 (62.2)	45 (37.8)		
**Having a sibling or other relative a physician**			**x**^**2**^ **=** 0.12	0.726
Yes	140 (72.2)	54 (27.8)		
No	161 (70.6)	67 (29.4)		
**Having a friend**,** or a family member living in Egypt**			**x**^**2**^ **=** 5.51	0.019*
Yes	228 (74.5)	78 (25.5)		
No	73 (62.9)	43 (37.1)		
**Place of Accommodation**			**x**^**2**^ **=** 0.012	0.914
University dorms	46 (70.8)	19 (29.2)		
Private place	255 (71.4)	102 (28.6)		

x^2^ = chi-square test value, * Significant p value

The categories with the greatest scores were: perceived discrimination and non-specific concerns; whereas the category with the lowest values was: guilt. The cut-off level of 104 was used to calculate the prevalence of AS among the studied group. Current study results showed that the majority of the study participants (71.3%) was equal to or below the warning sign of acculturative stress (above 109), i.e. 95% CI (66.75-75.60%). Moreover, 28.7% of the study participants were above the warning sign, i.e. 95% CI (24.63-33.49%). Table 4 shows that being a female; being from non-Arab coteries; and not having a relative in Egypt were the most significant factors affecting the occurrence of acculturative stress (*p* = 0.007, 0.009 and 0.019, respectively).

**Table 5:** Linear Regression describing factors affecting ASSIS total score

Factor (ref.)	Standardized Coefficients Beta	*P*-value	95% confidence interval for B	
			Lower bound	Upper bound
Age	0.016	0.718	-0.963	1.397
Gender (Male)	0.084	0.067	0.266	7,71
Nationality (Arab)	0.22	≤ 0.001*	5.78	15.62
Was one of your siblings or other relatives a physician? (No)	-0.011	0.807	-4.321	3.364
Do you have a friend enrolled in the same university or living in Egypt? (No)	-0.001	0.975	-4.38	4.24
Total Student Adjustment to College Score	-0.082	0.092	-0.885	0.066
Total Language Deficiency Score	-0.193	≤ 0.001*	-0.539	-0.177
Total Academic Adjustment Score	-0.2	≤ 0.001*	-0.72	-0.25
Total comfortability of living in the accommodation place score	-0.181	≤ 0.001*	-0.901	-0.276
Place of Accommodation (University Dorms)	0.068	0.173	-1,816	10.089
Constant		≤ 0.001*	106.48	158.6
Adjusted r squared = 0.212, f test = 11.63, p value ≤ 0.001				

Table 5 shows that the factors significantly associated with acculturative stress total score were: nationality (β = 0.220, p value ≤ 0.001), total language deficiency score (β= -0.193, p value ≤ 0.001), total academic adjustment score (β= -0.200, p value ≤ 0.001), and total comfortability of living in the accommodation place score (β= -0.181, p value ≤ 0.001).

## Discussion

The purpose of the current study was to assess the degree of acculturative stress experienced by international students at the medical faculty and to determine the elements that contribute to this stress. The study featured 422 international students studying at the college of medicine, 303 of whom were Arab, and 119 of whom were not. The study findings demonstrated that non-Arab students as well as female international students are more vulnerable to acculturative stress. A statistically significant correlation was found between how much students adjusted to college, how comfortable they felt living there, how well their academic adjustment scores were, how much the students were proficient in the language, and whether they experienced an acculturative stress warning sign.

The current sample’s mean score on the Acculturative Stress Scale for International Students (ASSIS) (M = 97.4) was greater than the normative mean (M = 66.32) provided by the ASSIS’s authors [8].

This result was slightly higher than the mean score in a research done in Germany and China, where the mean total scores were (M = 95.05) and (M = 92.81), respectively [2, 17].

The mean ASSIS score in the current study was higher than the mean score in a previous study that measured the overall score of acculturative stress. This aforementioned study involved international students enrolled in a public medical university in Romania (M = 85.82). The mean score of (M = 96) was reported by another U.S. study [18].

When compared to other studies conducted in Malaysia, the findings of the current study were lower. On the 36-item ASSIS, the average worldwide score for acculturative stress was shown to be 114.13 [19].

Moreover, by contrasting the findings of this study with those of other studies, it was seen that international students at this university were experiencing more acculturative stress than those at many other universities across the globe. Notably, it should be mentioned that there has been a sudden rise in the enrollment of foreign students in recent years, which may have presented difficulties for the faculty. For that reason, this may explain the higher acculturative stress.

According to the current study, through having a deeper comprehension of these students’ academic difficulties, instructors and staff at universities would therefore be more able to recognize the needs of their students. Instuctors and staff members could then also offer helpful campus resources and services. In addition to meeting students’ academic needs, the institution also needs to be prepared to meet the students’ social and cultural needs.

Additionally, this study implies that the university hosting overseas students should make certain arrangements before they arrive at the foreign country [20].

The most notable finding was that in the current study, 28.7% of the participants were above the “warning sign” category (above 109) on the ASSIS, compared to only 12.87% of the participants who scored above the “warning sign” category on the ASSIS among international students in the U.S. [21]. This indicates that there is a need for psychological interventions. Moreover, only 2.5% exceeded the same score of 109 in Sandhu and Asrabadi’s sample [8]. Another study conducted to measure the acculturative stress level and find out its predictors among international students [22] reported that 22.4% of the participants scored higher than 109, which indicated alarming stress levels at the university of Nebraska, Omaha, U.S.A.

Governmental universities from all over Egypt receive thousands of international students every year. This fact requires conducting a nation-wide survey of international students to be more able to generalize study results.

According to this study’s results, the international students’ most common reported cause of acculturative stress at the study setting (mean ± SD = 26.05 ± 7.18) was non-specific concerns. As a result, language and cultural barriers are the main causes of stress. These non-specific concerns include: (a) one’s degree of language competency in the host nation; (b) social anxiety; and (c) worries about how other people perceive one’s culture and/or ethnicity. Medical students at Ain Shams University had previously reported in a qualitative study that a major issue they faced was the language barrier, which affected them both academically and in their day-to-day activities [23].

The current study’s findings agreed with those from a previous study conducted among international students attending a public university in Malaysia. Of these students, the majority were from South Asia (48.2%; India, Bangladesh, and Pakistan), South-East Asia (28.4%; Brunei and Indonesia), as well as the Middle East and Africa (20.2%; Egypt, Jordan, and Yemen) [19]. 27% of the students showed stress due to non-specific concerns.

These findings were different from the findings of an earlier survey, which indicated that the non-specific concerns had the second-highest mean score in international students studying in Germany. Students were mainly from Asia and Europe (42.6% and 34.5%, respectively) while around 23% were from Latin America and Africa [2].

Perceived discrimination ranked as the second most stressful factor in this study. This was in contrast to the findings of [11]. This study found that among 20 Pakistani students who were studying in China, the perceived discrimination subscale exhibited the most amount of variance (38%) in the overall score on the ASSIS when compared to other subscales.

A few possible explanations for this perceived discrimination include: cultural shock, status loss, and low self-esteem brought on by a lack of family support. These are the difficulties that most international students have when they move to a new cultural setting [11].

As for homesickness, it had the third-highest mean score in this study, which is in contrast to the findings of a German study [2]. The study discovered that the participants’ reported homesickness scores were the highest. The results of the current study were consistent with those of [4], in which homesickness had the third highest mean score.

Another element, which contributed to the acculturative stress that many of the participants in Talwar et al.‘s study experienced, was homesickness. Since they had been removed from the safety of their homes, neighborhoods, and cultures, international students frequently experienced extreme loneliness in their new surroundings [19].

According to the results of a study assessing the social and academic adjustment of international Saudi students attending Australian universities, the participants underwent some forms of cultural stress, particularly during their first few semesters. It was discovered that the primary challenging aspects were discrimination, homesickness, and loneliness [24].

Consistent with the current findings, Akhtar and Kröner-Herwig observed in another study that among international students studying in Germany, guilt and fear were the least reported stressors. Most of the international students in Akhtar’s study did not hold themselves responsible for moving away from their families and home countries to live a different lifestyle in Germany. The majority of the students stated that despite coming from a diverse cultural background, they did not fear for their personal safety [2].

Furthermore, acculturative stress was linked to numerous factors, according to an earlier research. Language barriers, cultural diversity, personality, and social inclusivity are a few of these issues. Age, gender, and language proficiency had the most documented differences [2, 4, 25].

No significant association was found between the person’s age and the level of acculturative stress, which was in accordance with previous studies [13, 22].

Compared to male students, female students reported higher levels of acculturative stress; they were particularly more likely to experience higher degrees of fear and homesickness (Supp. Table 1). This result contrasts with the findings of a prior study, which indicated that gender identity was not an important predictor of the observed variations in the degree of acculturative stress [2]. This study contradicts some other researches that were conducted in the U.S. and Malaysia which found male students to have much higher levels of acculturative stress than their female counterparts [19, 26].

According to a previous study [4], being a female raises the acculturative stress level. Similarly, it was found that Kashmiri college students who migrated to Bhopal had higher level of stress reported by the female students of the study. This may be because of their less exposure to living and studying in another culture [27].

There was no significant difference in the degrees of homesickness between male and female international students in a study conducted at Midwestern University in the U.S.A. The study was done to examine the effects of adjustment issues, homesickness, and perceived discrimination on the psychological well-being of international faculty students [28].

Additionally, it has also been discovered that the perceptions of acculturative stress among international students are linked to their nation of origin. When non-Arab students were compared to Arab students, the former expressed more fear, discrimination, perceived hostility, and acculturative stress (Supp. Table 2). Similar to another study done in Romania, international students of Romanian descent likely had a low stress level because they had retained some form of host-country interaction from their parents or grandparents (food, language, relationships, family members, etc.) [4].

Furthermore, international students from non-European nations reported higher levels of acculturative stress than students from European nations in a German study. The results were noted in several publications [2, 17, 22] and showed that foreign students enrolled in American universities reported a noticeably higher degree of acculturative stress than students from European nations did [2, 17].

However, in contrast to results in the present study, [4] revealed that participants from non-European and European countries of origin did not significantly differ in terms of their total level of acculturative stress.

According to the results of the current study, having friends and family in the host nation reduced stress. This study is similar to another one, in which it was found that the presence of friends and family reduced the rate of nonspecific concerns, perceived discrimination, hate/rejection, and acculturative stress [4].

The results showed that students who scored the lowest in other subscales (i.e. food, climate, transportation, and comfortability in accommodation) had a high level of acculturative stress. These subscales also correlated negatively with an ASSIS total score, and some of its subscales: perceived discrimination, homesickness, fear, stress due to change/culture shock, and guilt (p value ≤ 0.001).

Previous research has shown that there were negative correlations between these characteristics and acculturative stress and some of its subscales. This is consistent with the findings of this study [4].

As students leave from their home country to the one in which they will be studying, they have to adopt to and integrate into the traditions and culture of their host nation. For international students, adjusting to the local food, climate, housing, and language is a major challenge.

Regarding the comfortability of living in the accommodation place, students with the lowest score had high levels of acculturative stress. The total comfortability of living subscale was correlated negatively with the total acculturative stress scale (*r*= -0.257, P value = ≤ 0.001). This finding was similar to one in another study in Romania [4].

It has been previously reported that international students frequently struggle with language, and that these difficulties might make it challenging for the students to socialize with their peers. In the current study, the ASSIS total score and the majority of its subscales showed a negative correlation with the degree of comfortability when using English in academic settings: stress due to change/culture shock, and non-specific concerns (p value ≤ 0.001).

The findings of a research assessing the academic and cultural adjustment of international Saudi students enrolled in Australian universities showed that the majority of the challenges faced by Saudi students relate to expressive and written language. Because of their poor language proficiency, respondents said they had to spend more time studying or completing their assignments [24].

Regarding the Arabic language, which is the official language in Egypt, 9.7% of the study participants did not speak Arabic, and most of them expressed that they felt stressed when communicating in Arabic. The current study had a qualitative phase that was separately published [29]. International students who participated in this phase indicated a major concern regarding language. The majority of non-Arab students stated that the most challenging obstacles they encountered in Egypt were related to language. The majority expressed concerns regarding their inability to communicate with patients and therefore missing out on the actual medical education experience. Even some Arab students indicated that they had some difficulties with different Arabic dialects.

In a similar study in Romania, the more comfortable the students who had Romanian origins feel communicating in Romania [4], the less they score on the stress due to change/culture shock subscale of the ASSIS. In other words, language can have an impact on international students’ academic demands. Previous research has shown that international students struggled to adapt because of their relatively low English language proficiency. Additionally, there is a significant correlation between language demands and academic needs because foreign students may find it difficult to participate in class discussions due to these needs. As a result, this can also hinder their ability to understand lectures [18].

A committed and focused student can increase his/her English proficiency. However, a “culture shock” of this kind can be severe and last for a long time. For that reason, it can be a difficult task [21] for a student from a vastly different cultural background.

The degree to which a student can effectively handle many academic demands, including motivation, application, performance, and environment satisfaction, is known as “academic adjustment”. People find it difficult to regulate themselves in order to stay in balance within their new academic setting and meet the university’s new academic requirements [30].

It is not only required from the host university to cover the students’ academic needs but also the social ones. Organizing social gathering events that include students from the host country and other recreational activities is strongly needed to help international students better understand and quickly merge into the new culture.

In a previous study in South Korea, perceived hate/rejection, and cultural shock negatively affected academic adjustment. Likewise, in this study, academic adjustment score correlated negatively with the ASSIS total score (*r*= -0.338, P value ≤ 0.001) and its subscales [31].

It is not surprising that perceived fear has an impact on how well students adjust academically and socially. Examples of this fear include: fear of failure, fear of making mistakes, fear of non-native accents, fear of the unknown, and fear of uncertainty in the new Egyptian educational and sociocultural context [31].

In the present study, a lower score in the student’s adjustment to the faculty tool was associated with a higher level of acculturative stress. This indicates that the student adaptation to the faculty is crucial to manage the process of acculturation. The total student’s adjustment to the faculty subscale was associated negatively with the total ASSIS score and most of its subscales: perceived discrimination, homesickness, fear, stress due to change/culture shock, and non-specific concerns (p value ≤ 0.001).

In a research conducted in Pakistan, the degree of social adjustment and self-esteem of international students were both examined. In terms of social aspects, the findings indicated that stress related to culture was experienced by international students. These outcomes line up with those of another study [32].

It is important to have a holistic view of the international students’ lives. There should be more research done to examine the students’ psychological status. That is because anxiety or depression might have an additional effect on acculturative stress.

According to these data, it can be inferred that acculturative stress among international students is associated with multiple factors, which are the nationality, language deficiency, academic adjustment, and comfortability of living in the accommodation place. That being said, the country of origin (which is defined in terms of nationality or language deficiency) is the most important stressing factor. In a similar study, it was found that family support, year of study, and difficulties in comprehending lectures were significant predictors of acculturation stress among students [19].

### Strengths and limitations of the study

The current study sheds some light on international medical students, a group that has not received much attention in research regarding acculturation and acculturative stress. Having said that, this study has its limitations. First, the study did not assess the students’ mental health. The authors are aware that persistent physical issues, depression, or worry can all hasten the process of acculturative stress. Moreover, data from only one medical university was used in the study sample. Therefore, it is necessary to do more research using various sample techniques. Since the study was cross-sectional, it cannot establish a cause-and-effect relationship between all the variables. It is important to take caution when generalizing study results due to these aforementioned limitations.

## Conclusion

The phrase “acculturative stress” is frequently employed to describe the unique challenges associated with immigration, as it may be interpreted as a stressful event. A multitude of factors, such as psychological strain from exams, social pressure, lengthy faculty appointments, and difficult course material, are additional stressors that experienced by medical students. Numerous elements are related to the experience of acculturative stress, as evidenced by earlier studies on its risk factors. Among these are: barriers related to language, cultural diversity, personality, and social inclusion.

The results of the current study indicated that the major stressors of acculturative stress among international students at the Faculty of Medicine included: the non-specific concerns (language and cultural concerns), homesickness, and perceived discrimination. As for the least reported stressors, they included: guilt and fear. Acculturative stress among the studied groups is influenced by several factors, including nationality, English and Arabic language proficiency, academic adjustment, and comfort level in living. However, the most significant stressor is the country of origin, which is defined in terms of nationality or language proficiency.

As a result, resources that sustain the long-term development of international students throughout their educational journey must be made available to a multicultural setting in order to support and retain these students. Students can receive support during their academic journey and career from a variety of sources. These sources include personal ones as the students’ psychological traits and internal drive. The sources also include other external ones as family, friends, the academic community, social media, or professional staff working in the university field.

## Electronic supplementary material

Below is the link to the electronic supplementary material.

Supplementary Material 1

## Data Availability

The datasets generated and/or analysed during the current study are not publicly available but are available from the corresponding author on reasonable request.

## Abbreviations

ASSIS: Acculturative Stress Scale for International Students
SD: Standard Deviation
SPSS: Statistical Package for the Social Science
U.S.: The United States
