# Supplementary material for: Factors associated with acculturative stress among international medical students in an Egyptian university
Source: BMC Med Educ. 2024 Sep 15;24:1009. doi: 10.1186/s12909-024-05947-5 (PMC11403887; doi:10.1186/s12909-024-05947-5)
Supplement: Supplementary file 1 — Supplementary Material 1 [file 12909_2024_5947_MOESM1_ESM.docx]

# Supplementary

Table 1 Relation between gender and acculturative stress total score and subscales (N=422):

| **Subscale (minimum- maximum)** | Male | Female | **t test** | **P value** |
| --- | --- | --- | --- | --- |
|  | **mean ± SD** | **mean ± SD** |  |  |
| **Perceived discrimination (8-40)** | 19.94 ± 5.72 | 20.81 ± 7.1 | -1.36 | 0.175 |
| **Homesickness (4-20)** | 13.96 ± 3.62 | 15.26 ± 3.34 | -3.79 | ≤ 0.001* |
| **Perceived hate/rejection (5-25)** | 12.84 ± 4.25 | 12.97 ± 4.69 | -0.31 | 0.76 |
| **Fear (4-20)** | 8.59 ± 3.30 | 9.79 ± 3.57 | -3.61 | ≤ 0.001* |
| **Stress due to change/culture shock (3-15)** | 9.02 ± 2.74 | 9.53 ± 2.79 | -1.89 | 0.060 |
| **Guilt (2-10)** | 5.09 ± 2.17 | 5.31 ± 2.18 | -1.06 | 0.292 |
| **Non-specific concerns (10-50)** | 25.45 ± 6.99 | 26.78 ± 7.41 | -1.9 | 0.058 |
| **Total score (36-180)** | 94.89 ± 21.23 | 100.46 ± 22.22 | -2.63 | 0.009* |

Table 2 : Relation between nationality and acculturative stress total score and subscales (N=422):

| **Subscale (minimum- maximum)** | Arab country | Non-Arab country | **t test** | **p value** |
| --- | --- | --- | --- | --- |
|  | **mean ± SD** | **mean ± SD** |  |  |
| **Perceived discrimination (8- 40)** | 19.46 ± 5.94 | 22.61± 6.94 | -4.37 | ≤ 0.001* |
| **Homesickness (4- 20)** | 14.95 ± 3.34 | 13.51 ± 3.87 | 3.8 | ≤ 0.001* |
| **Perceived hate/rejection (5- 25)** | 12.34 ± 4.25 | 14.33 ± 4.62 | -4.21 | ≤ 0.001* |
| **Fear (4-20)** | 8.80 ± 3.42 | 9.97 ± 3.50 | -3.16 | 0.002* |
| **Stress due to change/culture shock (3- 15)** | 9.26 ± 2.75 | 9.23 ± 2.83 | 0.113 | 0.910 |
| **Guilt (2-10)** | 5.34 ± 2.14 | 4.81 ± 2.22 | 2.27 | 0.024* |
| **Non-specific (10-50)** | 25.68 ± 6.95 | 27.00 ± 7.73 | -1.7 | 0.09 |
| **Total score (36-180)** | 95.81 ± 20.58 | 101.45 ± 24.37 | -2.41 | 0.017* |

| **a** |  |
| --- | --- |
|  |  |

**Figure 1 : Correlations between ASSIS and different scales measured in the study**

**Table 3 : Means, Standard Deviations, and Cronbach’s Alpha Levels for the Seven Subscales of the ASSIS**

| **Subscale name** | No. of items | Mean | SD | α |
| --- | --- | --- | --- | --- |
| **Perceived Discrimination** | 8 | 20.3 | 6.4 | 0.819 |
| **Homesickness** | 4 | 14.5 | 3.5 | 0.707 |
| **Perceived Hate** | 5 | 12.9 | 4.4 | 0.806 |
| **Fear** | 4 | 9.13 | 3.5 | 0.747 |
| **Stress Due to Change/Culture Shock** | 3 | 9.25 | 2.7 | 0.61 |
| **Guilt** | 2 | 5.19 | 2.17 | 0.63 |
| **Miscellaneous** | 10 | 26.05 | 7.19 | 0.774 |
| **Total score** | 36 | 97.4 | 21.8 | 0.913 |
